# Estimation of Lactate Thresholds, Aerobic Capacity and Recovery Rate from Muscle Oxygen Saturation in Highly Trained Speed Skaters and Healthy Untrained Individuals

**DOI:** 10.3390/jcm13175340

**Published:** 2024-09-09

**Authors:** Kinga Rębiś, Andrzej Klusiewicz, Barbara Długołęcka, Paweł Różański, Karol Kowieski, Tomasz Kowalski

**Affiliations:** 1 Department of Physiology, Institute of Sport–National Research Institute in Warsaw, 01-982 Warsaw, Poland; 2 Faculty of Physical Education and Health, Józef Piłsudski University of Physical Education in Warsaw, 00-968 Biała Podlaska, Poland; andrzej.klusiewicz@awf.edu.pl (A.K.); barbara.dlugolecka@awf.edu.pl (B.D.); pawel.rozanski@awf.edu.pl (P.R.); karol.kowieski@awf.edu.pl (K.K.)

**Keywords:** exercise, physical fitness, cardiopulmonary exercise testing, near-infrared spectroscopy, anaerobic threshold

## Abstract

**Objective:** The main objective of this study was to compare lactate thresholds and aerobic capacity from a graded-intensity exercise test (GXT) for near-infrared spectroscopy measurements in healthy, untrained individuals and highly trained athletes. **Methods:** This study included 29 untrained students (13 females) and 27 highly trained speed skaters (13 females). A maximal effort GXT was performed on a cycloergometer. The lactate-based aerobic and anaerobic thresholds, and the corresponding thresholds for muscle oxygen saturation (SmO_2_), were determined. **Results:** The power values determined for all thresholds were significantly higher in female and male speed skaters compared to male and female college students. SmO_2_ at anaerobic thresholds was significantly lower in female speed skaters than in female students. Both female and male skaters showed greater changes in SmO_2_ after the GXT compared to students. The recovery did not significantly differ between groups within gender. There was a significant positive correlation in females between the rate of muscle reoxygenation and VO_2_max power (r = 0.610). In speed skaters, the rate of muscle reoxygenation was not significantly higher than students and correlated positively with VO_2_max (r = 0.449). **Conclusions:** The SmO_2_ at the exercise thresholds, during and after maximal exercise, depends on the training status of the individual. The participants with a higher physical fitness level showed greater decreases in ΔSmO_2_ at the AT level, as well as after maximal exercise. SmO_2_ corresponding to the well-established exercise thresholds may be applied to guide training prescription. The rate of muscle reoxygenation after a GXT was also dependent on the aerobic capacity of the participants.

## 1. Introduction

Over the past few years, wearable devices that apply near-infrared spectroscopy (NIRS) to assess the oxygenation (muscle oxygen saturation, SmO_2_) level of myoglobin in the muscle cytoplasm, and hemoglobin in the blood vessels of muscle, have been introduced into scientific research and training practise [1,2,3]. As myoglobin oxygenation is expected to remain almost unchanged during exercise, any alteration in myoglobin and hemoglobin signals should mainly reflect changes in hemoglobin [4]. SmO_2_ reflects the dynamic balance between oxygen (O_2_) supply and O_2_ demand, mainly at the superficial level of muscle [3], whilst providing real-time information on oxygen utilization during exercise [5]. It is a local measurement reflecting differences or changes in SmO_2_ during exercise, depending on NIRS placement [6], and the NIRS device can be worn in laboratory or field settings to improve ecological validity.

In the sport of speed skating, modern oximeters allow for on-body measurements of SmO_2_ in the laboratory [7,8] and in natural training conditions [9,10], and these are becoming useful in evaluating training effects [11,12]. The measured indices may be displayed in real time, similar to heart rate recordings, on a smartphone, tablet or computer. Our previous research has confirmed that NIRS offers a simple, safe and fast way to both assess maximal exercise capacity [13] and monitor aerobic training load [14]. Other laboratories have confirmed the utility of NIRS for the determination of intensity zones from an incremental load test [15,16], and for establishing the anaerobic threshold (AT cut-off point), based on the dynamics of SmO_2_ changes during exercise [1,15,17,18,19,20]. The aforementioned studies are of significant practical importance, as they confirm the efficacy of a non-invasive, wearable sensor for evaluating training intensity and selecting exercise loads [21].

From a practical point of view, establishing one’s anaerobic threshold (AT) is particularly important for the assessment of aerobic capacity, as well as the selecting of individualized training-intensity zones [22]. However, Jamnick et al. [22] identified more than 25 different methods of determining threshold load, potentially reducing the translation and interpretation of these measures in sport. The identification of the threshold for 2 and 4 mmol blood lactate concentration, or the modified Dmax threshold (ModDmax) and lactate threshold (LT), is easy to determine using available software [23]. The lactate thresholds discussed are relatively easily determined and widely applied in different sport settings [22,24]. Therefore, we investigated the exercise thresholds and associations with NIRS-derived SmO_2_ levels. The approach may be particularly useful in speedskaters, as restricted blood flow during major parts of the skating stroke cycle influences the measured lactate concentrations [25,26]. Traditionally, sports scientists and coaches draw conclusions regarding whole-body lactate dynamics based on lactate concentrations in capillary blood. However, in the case of speedskating and observed deviation from the normal relationship between local and whole-body lactate concentrations, the validity of traditional lactate-derived exercise thresholds was not thoroughly established [25]. Therefore, novel methods of intensity monitoring may play a crucial role in on-ice and inline speedskating. Despite this, research on NIRS application in speedskating remains limited.

Previous studies have described the SmO_2_ responses at LT levels in representative groups of healthy, untrained individuals and highly trained athletes, separately; however, the literature analyzing differences between these groups remains scarce. Also, limited research has evaluated the utility of muscle oxygenation across a graded exercise test (GXT) and compared the effect of training (or physical fitness) status. Moreover, muscle reoxygenation after exercise cessation may inform recovery effectiveness and training status [27]. Thus, the main aim of this study was to compare the aerobic and anaerobic thresholds based on SmO_2_ levels in healthy, physically active individuals and highly trained athletes. An additional goal of this study was to establish whether the SmO_2_ during, and after, a ramp-incremental test is contingent on training status and its association with well-established performance variables. The hypothesis of this study was that the level of physical fitness is associated with SmO_2_ at the aerobic threshold, AT, maximal deoxygenation levels and muscle reoxygenation rate, independently of gender.

## 2. Materials and Methods

This study included 29 students from the Academy of Physical Education and 27 Polish speed skaters, who were representing the National Team at a junior or senior level. The total sample size was calculated with G * Power (v3.1.9.7) and was 52 for two-tailed *t*-tests for independent means (effect size = 0.8, a = 0.05, power = 0.8) and 46 for multiple linear regression (effect size = 0.5, a = 0.05, power = 0.8, number of predictors = 5). The characteristics of the participants are shown in Table 1. The speed skaters (males and females) were significantly younger, taller and possessed a higher body mass, compared to male and female students, respectively. The speed skaters were also characterized by a significantly lower BMI, with the students presenting a mean BMI value (25.6 ± 3.4 kg/m^2^) that indicated that they were slightly overweight.

This study was conducted in cooperation with the National Speed Skating Association during the preparatory period of an annual training plan. The research outlined was conducted according to the guidelines of the Declaration of Helsinki and its later amendments, with approval no. KEBN-22-71-KR (14 March 2022) from the Research Ethics Committee of Institute of Sport–National Research Institute, Warsaw, Poland. The participants chose to partake voluntarily, and they were informed about the right to withdraw at every stage of the study. All participants provided written consent before study commencement, and in the case of underage participants, such consent was obtained from their legal guardians.

### 2.1. Graded Exercise Test

**Figure 1:**
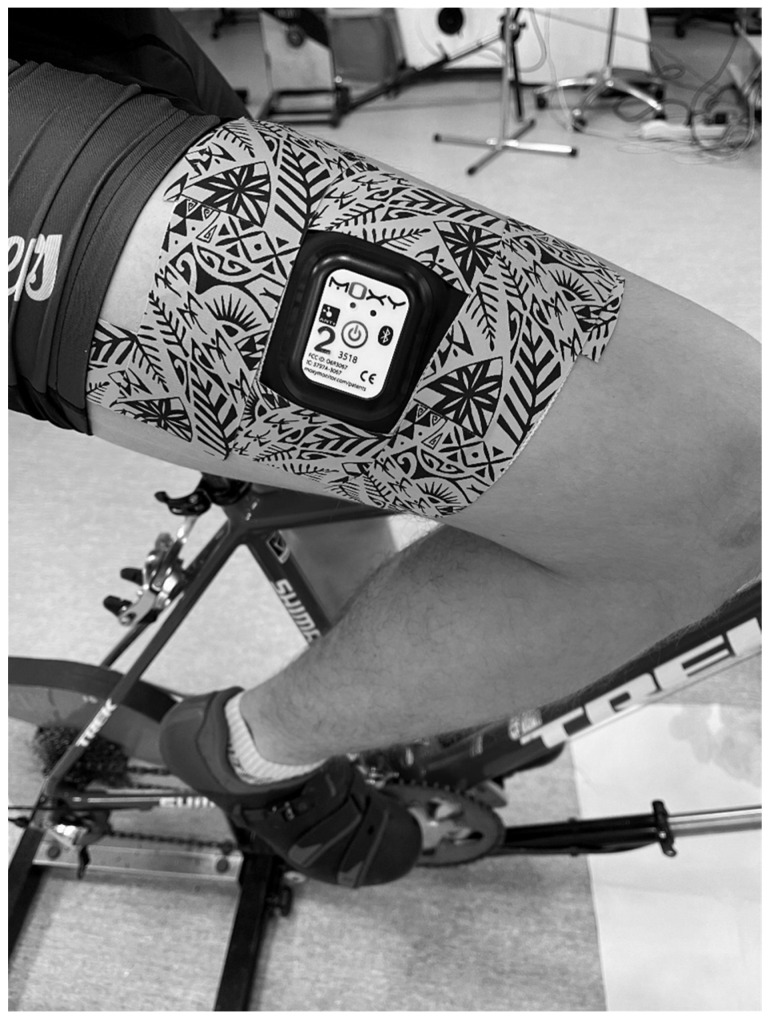
Location of the MOXY monitor on the vastus lateralis muscle belly.

A maximal-intensity GXT was performed on a bicycle ergometer with measurements of gas exchange, heart rate, the SmO_2_ of the vastus lateralis muscle and blood lactate carried out at regular intervals before and after exercise. Figure 1 shows the placement of the NIRS sensor.

The GXTs were performed using the Cortex MetaMax B3 (Cortex Biophysik GmbH, Leipzig, Germany), the breath-by-breath method and a cycloergometer. The gas analyser was previously demonstrated as a reliable tool for measuring oxygen uptake [28].

The Lode Corival ergometer (Lode B.V., Groningen, The Netherlands) was used in the student group, and the Cyclus II Ergometer (RBM, Leipzig, Germany) was used in the speed skater group. The elite speed skaters used a cycle ergometer based on an individual setting of the cyclist’s bicycle frame to achieve optimal conditions for the test task. The testing equipment had been calibrated according to the manufacturer’s instructions.

The participants underwent an incremental step test consisting of 3 min efforts performed continuously. The test commenced at 30–70 W, and the load increased for 30–50 W with every 3 min step, which was individually adjusted for sex, body mass and fitness status to achieve possibly similar testing times for all the participants (about 17–18 min). The participants were instructed to continue their effort until total exhaustion. After completing both tests, the participants continued pedalling for active cool-down (light exercise at a load of 0.5 W/kg performed for 3 min after the completion of the test), aimed at avoiding orthostatic shock. 

During the GXT, heart rate (HR) was continuously recorded using the H9 Heart Rate Sensor (Polar Electro Oy, Helsinki, Finland). Blood samples were taken from the fingertip during the last 15 s of every step, immediately after and 3 min after the cessation of the test. Each sample was collected in a 20 μL capillary tube by the laboratory technician. Blood lactate concentration was determined with the Super GL2 analyzer (Dr. Müller Gerätebau GmbH, Freital, Germany) immediately after the testing procedures.

### 2.2. Aerobic and Anaerobic Thresholds, Maximal Aerobic Power

Aerobic and anaerobic thresholds (2 and 4 mmol, modified Dmax and lactate threshold-LT), and the corresponding threshold SmO_2_ values, were derived from the application described by Newell et al. [23]. The aerobic (2 mmol) and anaerobic (4 mmol) thresholds were determined by the interpolation of the power output values and SmO_2_ registered at the 2 or 4 mmol/L blood lactate level. The criteria for determining modified Dmax were as follows: the performance of a minimum of 5 exercise loads of full duration (3 min) and a final blood lactate concentration of more than 8 mmol/L. Maximal aerobic power (*MAP*) was calculated as a proportion of time and power achieved in the last executed bout of the GXT, according to Kuipers et al. [29]. The equation for this calculation is presented below:

$$MAP=P_{LFE}+(\frac{T_{LE}}{T_{St}}\times \left.\Delta P\right)$$

*MAP*—maximal aerobic power; *P_LFE_*—power of the last fully executed step; *T_LE_*—time executed in the final step; *T_St_*—time of the last step; Δ*P*—increase in power between the last two steps.

### 2.3. Maximal Oxygen Consumption

Maximal oxygen uptake (VO_2_max) was defined as the highest amount of oxygen consumption over a 30 s period during exercise testing. The maximal intensity of exercise necessary for the estimation of VO_2_max was defined by the following criteria: the present oxygen uptake plateau (growth < 100 mL·min^−1^ in oxygen uptake despite an increase in workload), the post-exercise blood lactate concentration > 8 mmol/L, the respiratory exchange ratio (RER) > 1.1 and the attainment of the age-adjusted maximal heart rate, expressed as HRmax = 220-participant age. If at least two of the above criteria were met during the exercise, the attained effort and oxygen uptake were regarded as maximal.

### 2.4. Measurements of Muscle Oxygen Saturation

During the GXT, a NIRS device (Moxy Monitor; Fortiori Design LLC, Hutchinson, MN, USA) was placed on the vastus lateralis (VL) muscle that is active during cycling. The Moxy monitor is a continuous wave near-infrared spectroscopy sensor. It uses a new type of algorithm that is based on Monte Carlo modelling. The device has two detectors spaced at 12.5 and 25 mm from the emitter, and uses 4 wavelengths of 680, 720, 760 and 800 nm. The default sampling rate cycles through the four wavelengths 80 times every 2 s and averages out the readings for an output rate of 0.5 Hz. The device was placed approximately 15 ± 2 cm above the proximal border of the patella on the vastus lateralis muscle belly and was fixed to the right limb with a dark 7.5 cm flexible tape by the same person (Figure 1).

**Figure 2:**
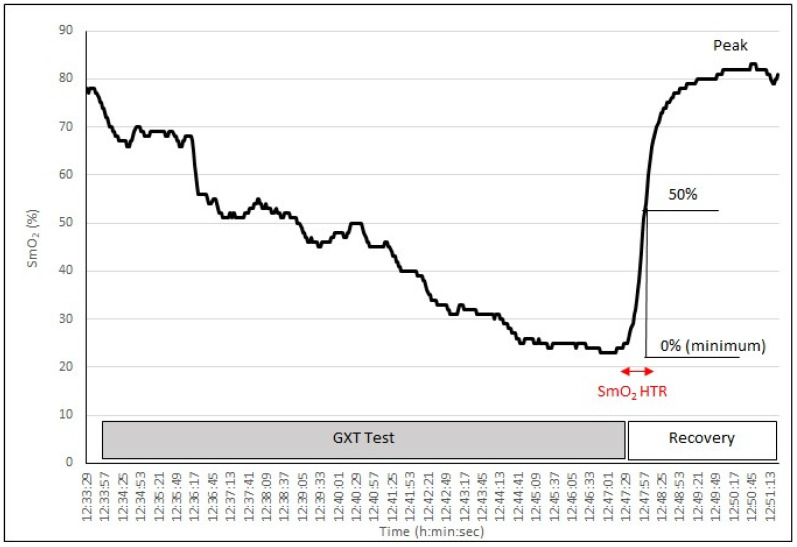
Method of evaluating the time for 50% recovery muscle oxygen saturation (SmO_2_ recovery half-time, SmO_2_HTR) after the maximal graded exercise test (GXT) for one male student (based on individual data from the present study, compiled according to the guidelines from Nagasawa, 2013).

The SmO_2_ was recorded at rest, during exercise and recovery. SmO_2_ measurements taken 2 min before the GXT were taken as baseline values. The differences in SmO_2_ (ΔSmO_2_) between baseline and minimal SmO_2_ during exercise were also calculated. The reoxygenation rate after the GXT was evaluated as the half-time required for SmO_2_ recovery (SmO_2_ Δ Time) [27,30], presented in Figure 2.

For other metrics, the SmO_2_ data were averaged from measurements made 2.0 min before testing (SmO_2_ rest), during and after the test with 2-s intervals (the default time interval in the accompanying Moxy software, Version 1.4.4). Data were recorded for the minimum SmO_2_ (2 s at end of the test) (minimum, SmO_2_ min) and over 2 s at recovery (maximum, SmO_2_ max). The SmO_2_ min after the completion of the exercise was defined as 0%, and the SmO_2_ max of the recovery phase was defined as 100%. SmO_2_ Δ time was then defined as the time from the completion of the exercise to the time to reach 50% SmO_2_Max [27]. SmO_2_ recovery rate was calculated according to the following formula:

SmO_2_ Recovery Rate (%/s) = 50% SmO_2_Max (%)/SmO_2_ Δ Time (s)

SmO_2_ threshold values were determined by averaging measurements at 30 s intervals for the respective exercise loads.

### 2.5. Statistical Analyses

All statistical analyses were conducted with Statistica 13.0 software (Statistica 13, TIBCO Software Inc., Palo Alto, CA, USA). The normal distribution of variables was examined using the Shapiro–Wilk test. The differences between groups in demographic data, oxygenation and performance parameters were assessed using a Student’s *t*-test or the Mann–Whitney U-test for variables with a non-normal distribution. The effect size was calculated as Cohen’s d (where 0.2 represents a small effect size, 0.5 represents a medium effect size and 0.8 represents a large effect size) for Student’s *t*-tests or rank biserial correlation (r, where 0.1 represents a small effect size, 0.3 represents a medium effect size and 0.5 represents a large effect size) for Mann–Whitney U-tests. The relationships between the oxygenation and performance variables were tested using Pearson’s correlation coefficient (R). A coefficient of determination (R^2^) was also computed to aid interpretation. We used published criteria, as described by Evans [31], to describe the strength of the correlations; <0.20 as very weak, 0.20–0.39 as weak, 0.40–0.59 as moderate, 0.60–0.79 as strong and >0.80 as very strong. The level of statistical significance was set at *p* ≤ 0.05.

## 3. Results

The female and male speed skaters were characterized by a higher physical capacity in comparison to male and female students, based on significant differences between the groups in terms of MAP and VO_2_max (Table 2).

In all cases, power values for the aerobic and anaerobic lactate thresholds (in W and W/kg) were significantly higher in female and male speed skaters relative to male and female students (Table 3). SmO_2_ values at anaerobic thresholds expressed in % were significantly lower in female speed skaters (AT4: 32.7 ± 11.5, Dmax: 31.0 ± 9.2 and LT: 33.9 ± 9.6) than in female students (45.2 ± 11.1, 44.3 ± 10.3 and 45.6 ± 10.5, respectively). In speed skaters, the differences in question, although not statistically significant, showed a similar direction of change (Table 3).

**Figure 3:**
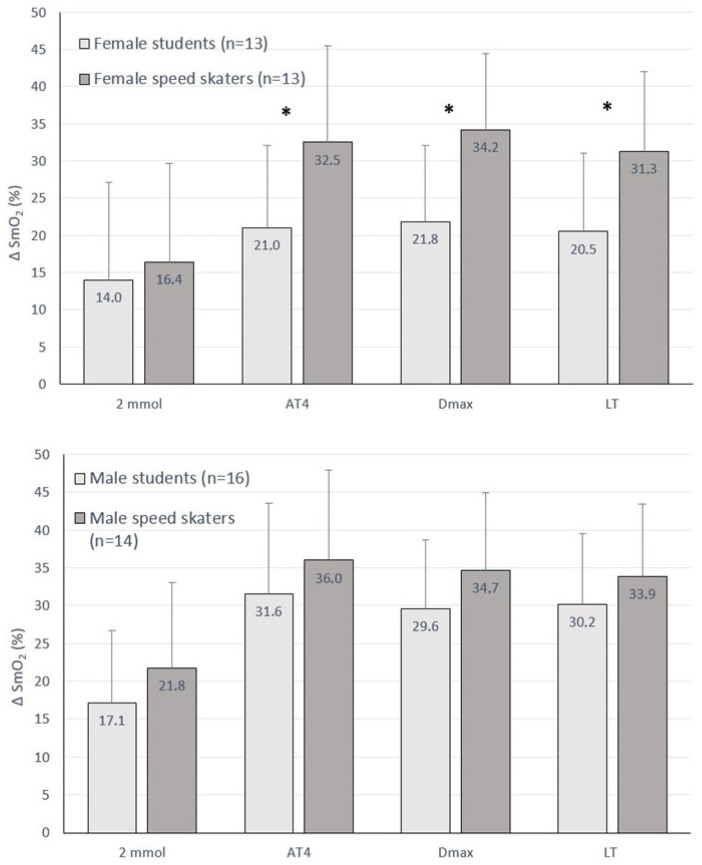
Muscle oxygen saturation (SmO_2_) at the aerobic (2 mmol) and anaerobic thresholds (4 mmol, Dmax, LT), expressed as Δ (SmO_2_ at Rest − SmO_2_ at the aerobic or anaerobic threshold) in the groups of students and speed skaters. *—difference (*p* < 0.05) between female students and female speed skaters.

The female speed skaters also showed significantly greater changes in SmO_2_ (differences between rest and anaerobic threshold SmO_2_ values in %) for lactate thresholds of 4 mmol, Dmax and LT (32.5 ± 13.0, 34.2 ± 10.2 and 31.3 ± 10.7) compared to female students (21.0 ± 11.1, 21.8 ± 10.3 and 20.5 ± 10.5) (Figure 3). Male skaters also showed greater ΔSmO_2_ values relative to male students, but these were not statistically significant.

**Figure 4:**
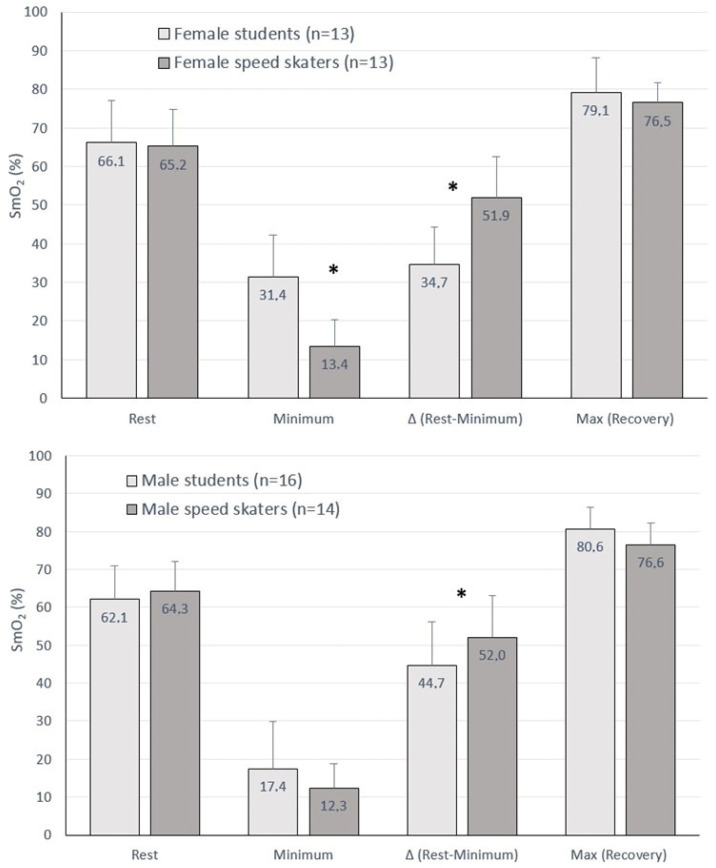
Muscle oxygen saturation (SmO_2_): at baseline, as exercise minimum values, differences between the baseline and minimum (Δ Baseline − Minimum) and the recovery maximum values during the maximal graded exercise test in the groups of students and speed skaters. *—difference (*p* < 0.05) between female students and female speed skaters, or male students and male speed skaters.

Both female and male skaters additionally showed a greater decrease in ΔSmO_2_ (differences between rest and minimal value) after the GXT, as compared to students (female 51.9 ± 10.7 vs. 34.7 ± 9.7% and male 52.0 ± 11.0 vs. 44.7 ± 11.5%, respectively) (Figure 4).

**Figure 5:**
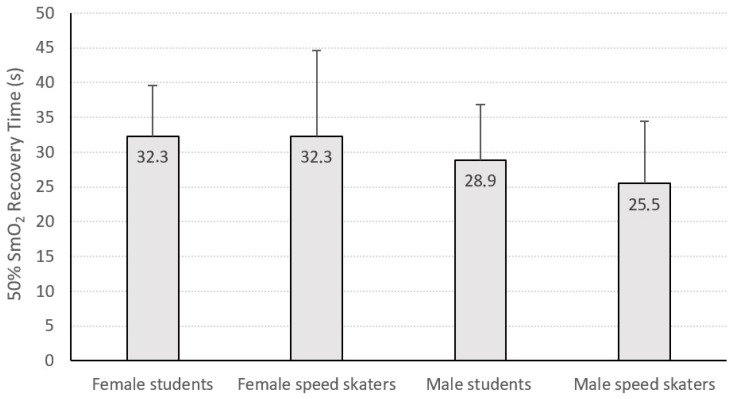
Time for the 50% recovery of muscle oxygen saturation (SmO_2_) in the groups of students and speed skaters.

The time to achieve 50% SmO_2_ in the recovery period (50% SmO_2_ recovery time) did not differentiate between female and male students and speed skaters (Figure 5).

**Figure 6:**
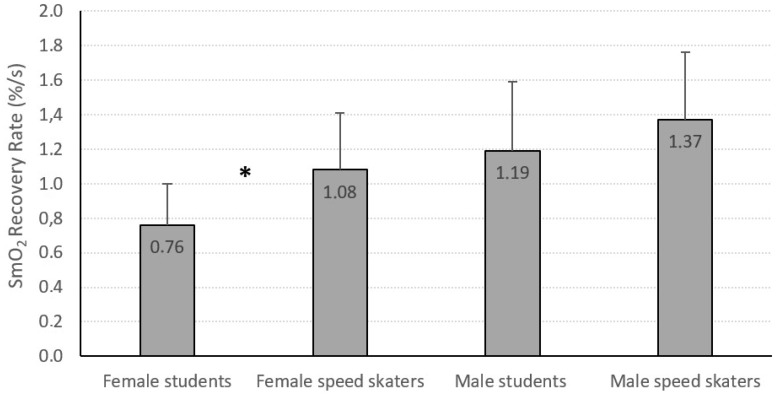
Values of SmO_2_ recovery rate in the groups of students and speed skaters. *—difference (*p* < 0.05) between female students and female speed skaters.

However, a higher recovery rate (SmO_2_ recovery rate) was observed in female speed skaters (1.08 ± 0.33 %/s) when compared to female students (0.76 ± 0.24 %/s) (Figure 6).

**Figure 7:**
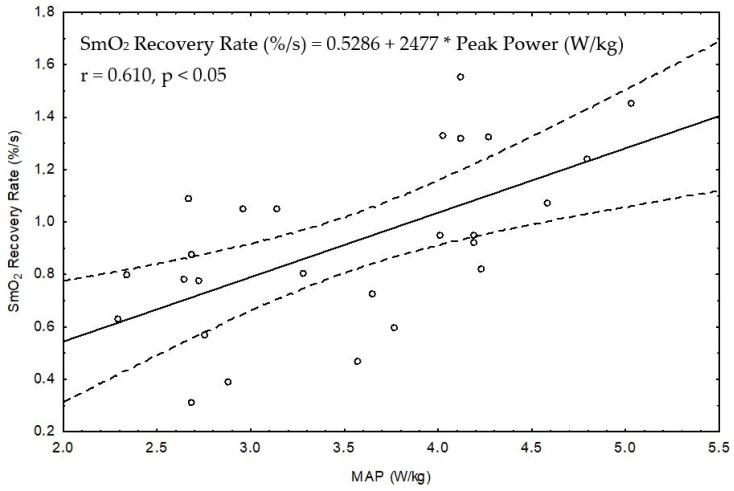
Statistically significant correlation between the SmO_2_ recovery rate and maximal aerobic power (MAP) in the combined groups of female students and speed skaters (n = 26).

Correlational analyses revealed a significant and strong relationship (r = 0.610, shared variance of ~37%) was observed in females between SmO_2_ recovery rate and peak power (W/kg) (Figure 7).

**Figure 8:**
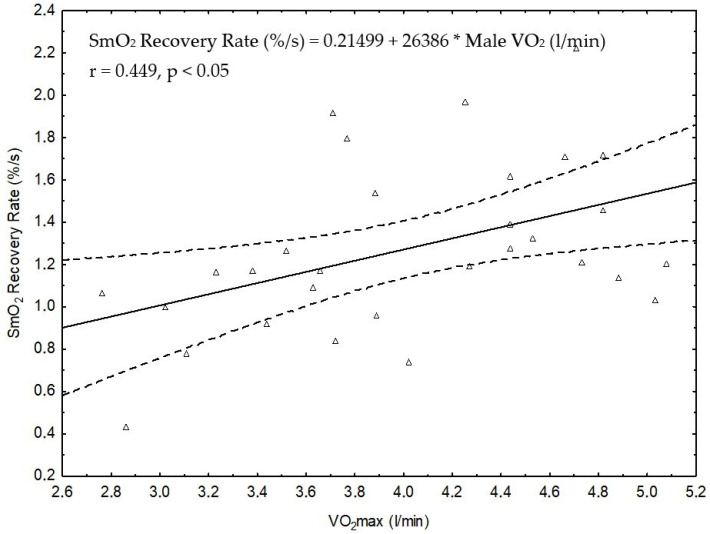
Statistically significant correlation between the SmO_2_ recovery rate and VO_2_max in the combined groups of male students and speed skaters (n = 30).

In male speed skaters, although the recovery rate was not significantly higher versus the student group (Figure 6), a significant and moderate relationship (r = 0.449, shared variance of ~20%) was observed between SmO_2_ recovery rate and VO_2_max (Figure 8). 

## 4. Discussion

The presented investigation reports multiple novel findings. The SmO_2_ at the AT, during and after maximal exercise, depends on the training status of the individual. Those participants with a higher physical fitness level showed greater changes in ΔSmO_2_ at the AT level, as well as after maximal exercise. The rate of muscle reoxygenation after a GXT was also dependent on the aerobic capacity of the participants.

In the available literature regarding the determination of AT values based on measurements of SmO_2_, studies have been conducted mostly among trained individuals [31,32,33,34] or in groups of recreationally trained individuals [19]. One of the main goals of our research was to compare aerobic and AT in groups with different training statuses, i.e., in highly trained speed skaters and untrained students. Blood lactate measurements are widely considered the gold standard to establish threshold values [35]. However, attempts to determine said thresholds based on non-invasive measurements are still of particular interest. In recent years, such studies have been undertaken using the SmO_2_ index [18,19,32,33,34]. Other work [19] has shown that both the maximum distance (Dmax) and modified maximum distance (mDmax) values should not be used to estimate the LT due to the unreliable detection of the SmO_2_ threshold from session to session. Similarly, in other studies [33], the ventilatory (VT) and tissue saturation index (TSI_T_) thresholds were not concurrent with the LT. In contrast to the cited study, Grassi et al. [36] reported a significant correlation between the onset of muscle deoxygenation and the onset of blood lactate accumulation (OBLA, a nominal La of 4 mmol/L). Similarly, Driller et al. [1] pointed out the agreement and practical usefulness of the AT determined in cyclists by a wearable NIRS device when measuring SmO_2_ compared to four traditionally used methods of determining the LT. Also, Borges and Driller [18] observed in runners no significant differences in load for the AT determined using the aforementioned NIRS sensor compared to five traditional methods, based on blood lactate determinations.

Consequently, there is still no consensus among authors on whether non-invasive, portable and easy-to-use NIRS measurement devices can adequately capture the aerobic–anaerobic transition by providing an equivalent to index to the LT that has been used for many years [37,38]. In our research, we used different lactate thresholds (“aerobic threshold”, “modf. Dmax”, “OBLA” and “LT”) to determine relevant values for SmO_2_. Although this method was benchmarked against blood lactate concentration measurements, it allowed us to use SmO_2_ values to monitor exercise intensity and training load. In the state-of-the-art literature, both the nominal values of SmO_2_ and the differences between resting and exercise SmO_2_ values (ΔSmO_2_) have been used [6,39]. Calculating ΔSmO_2_ allows for the large variation in resting values of SmO_2_ to be taken into account. Previously, the same way of expressing muscle oxygen saturation values was presented by Cayot et al. [19]. This method was also used for ΔSmO_2_ values, but was calculated in a slightly different way; it was used for the interpretation of data in studies of kayakers and canoeists [6].

In our study, a higher level of athletic training (female skaters vs. female students) was accompanied by a lower SmO_2_ value at the AT, when presented both on a nominal scale (Table 3) and described as ΔSmO_2_ (Figure 3). At the same time, the female skaters achieved higher power outputs at the AT. Speculatively, the SmO_2_ level at the AT may reflect relative training status, and NIRS measurements during submaximal efforts may offer a non-invasive marker of physical fitness. These findings are applicable to both aerobic and anaerobic training zones and apply to constant intensity efforts.

Notably, NIRS-derived thresholds are obtained locally, and over a small area of the activated muscle. However, both traditional methods of determining AT, based on blood lactate concentrations and ventilation threshold, reflect the body’s global response to a given effort. During exercise on the cycle ergometer, other muscles besides the quadriceps muscle group are working, which affects lactate production. It would be useful to test whether the NIRS threshold is dependent on the location of the monitor [32,33]. This question was answered by Batterson et al. [31], and it was confirmed that breakpoints in muscle O_2_ saturation rate (%/min) were not significantly different from LT1 or LT2 at any muscle sites. In response to maximal effort, larger decreases in SmO_2_ should be associated with higher levels of physical capacity (Figure 4). Previously, a similar relationship was identified by Okushima et al. [40] and Feldmann et al. [15], confirming that trainees with a higher VO_2Peak_ showed a greater decline in SmO_2_ during maximal exercise. Therefore, the oxygen uptake capacity between capillaries and skeletal muscle appears to be related to the aerobic capacity of the active muscle.

The selection of recovery duration to evaluate the reoxygenation rate of muscle has a notable practical implication. Our previous study in highly trained speed skaters [13] found that reoxygenation rate showed a significant positive correlation with VO_2max_ and exercise capacity in the GXT. In the current study, we also demonstrated a strong positive correlation between the rate of reoxygenation with peak power in the GXT (in females) and a moderate correlation with VO_2max_ (in males) (Figure 7 and Figure 8). These correlations offer further evidence that the rate of muscle reoxygenation after a GXT depends, in part, on the aerobic capacity of the participants. In related work, Ichimura et al. [41] noted a faster rate of muscle reoxygenation after a ramp test in those with higher VO_2max_ levels.

## 5. Conclusions

The SmO_2_ at the AT, during and after maximal exercise, depends on the training status of the individual. Those participants with a higher physical fitness level showed greater decreases in ΔSmO_2_ at the AT level, as well as after maximal exercise. SmO_2_ corresponding to the well-established exercise thresholds may be applied to guide training prescription. The rate of muscle reoxygenation after a GXT was also dependent on the aerobic capacity of the participants.

## 6. Limitations

Two types of cycle ergometers were used in this study. The skaters opted to use their own professional bicycle frames mounted on a Cyclus cycle ergometer to achieve optimal test results. In contrast, students performed the tests on a Lode Corival, which allows for precise positioning that is suitable for amateurs. Notably, the choice of ergometer model did not affect physiological exercise indicators such as HRmax, VO_2_max, Lactate PEAK, or SmO_2_. Finally, the size of the investigated population might be considered a study limitation. However, studies including elite athletes are subject to such constraints, as the investigated population remains small.

## Figures and Tables

**Table 1:** Basic characteristics (mean ± SD) of females and males in the examined group of students and speed skaters.

Females		
Variable/Group	Students (n = 13)	Speed Skaters (n = 13)
Age (years)	23.1 ± 1.0	18.5 ± 2.0 *
Body height (cm)	164.9 ± 4.7	168.2 ± 3.7 *
Body mass (kg)	60.7 ± 6.5	60.4 ± 3.6
BMI (kg/m^2^)	22.3 ± 3.7	21.3 ± 1.0
Training experience (years)	-	4.2 ± 1.5
**Males**		
	**Students** **(n = 16)**	**Speed Skaters (n = 14)**
Age (years)	23.8 ± 1.1	17.9 ± 2.2 *
Body height (cm)	181.1 ±7.1	182.0 ± 6.8 *
Body mass (kg)	84.0 ± 11.8	73.9 ± 6.5 *
BMI (kg/m^2^)	25.6 ± 3.4	22.3 ± 1.2 *
Training experience (years)	-	3.5 ± 0.9

*—statistically significant differences between female students and female speed skaters or male students and male speed skaters *p* < 0.05.

**Table 2:** Peak values (mean ± SD) of selected indices during the maximal graded exercise test in female and male students and speed skaters.

Females					
Variable/Group	Students (n = 13)	Speed Skaters (n = 13)	*p*-Value	Effect Size	
				Cohen’s d	Rank Biserial Correlation (r)
Test time (min: s)	16:51 ± 2:10	16:59 ± 1:51	0.87	0.06	-
MAP (W)	169 ± 22	254 ± 18 *	<0.001	-	1.0
MAP_rel_ (W/kg)	2.82 ± 0.37	4.04 ± 0.33 *	<0.001	3.67	-
VO_2_max (L/min)	2.28 ± 0.28	3.18 ± 0.26 *	<0.001	3.31	-
VO_2_max (mL/kg/min)	38.0 ± 3.7	52.1 ± 4.6 *	<0.001	1.68	-
Lactate _PEAK_ (mmol/L)	10.7 ± 1.7	13.1 ± 1.3 *	<0.001	1.61	-
**Males**					
**Variable/Group**	**Students** **(n = 16)**	**Speed Skaters (n = 14)**	***p*** **-Value**	**Effect Size**	
				**Cohen’s d**	**Rank Biserial** ** Correlation (r)**
Test time (min: s)	18:32 ± 2:17	18:05 ± 2:07	0.59	0.20	-
MAP (W)	258 ± 32	353 ± 45 *	<0.001	2.86	-
MAP_rel_ (W/kg)	3.10 ± 0.45	4.77 ± 0.43 *	<0.001	4.08	-
VO_2_max (L/min)	3.58 ± 0.54	4.53 ± 0.42 *	<0.001	3.10	-
VO_2_max (mL/kg/min)	42.9 ± 6.7	62.0 ± 4.8 *	<0.001	1.98	-
Lactate _PEAK_ (mmol/L)	11.8 ± 1.8	13.5 ± 2.3 *	0.03	0.83	-

MAP—absolute maximal aerobic power, MAP_rel_—relative maximal aerobic power; lactate _PEAK_—post-exercise lactate; *—difference (*p* < 0.05) between female students and female speed skaters, or male students and male speed skaters.

**Table 3:** Values (mean ± SD) of selected indices at the aerobic and anaerobic thresholds in female and male students and speed skaters.

Females					
Variable/Group	Students (n = 13)	Speed Skaters (n = 13)	*p*-Value	Effect Size	
				Cohen’s d	Rank Biserial Correlation (r)
P_2 MMOL_ (W)	68 ± 22	122 ± 35 *	<0.001	1.86	-
P_2 MMOL_ (W/kg)	1.14 ± 0.34	2.00 ± 0.64 *	<0.001	1.71	-
P_AT4_ (W)	114 ± 19	183 ± 21 *	<0.001	3.51	-
P_AT4_ (W/kg)	1.90 ± 0.29	3.04 ± 0.41 *	<0.001	3.248	-
P_DMAX_ (W)	112 ± 13	180 ± 22 *	<0.001	3.72	-
P_DMAX_ (W/kg)	1.88 ± 0.25	3.00 ± 0.45 *	<0.001	-	1.00
P_LT_ (W)	105 ± 12	169 ± 21 *	<0.001	-	0.99
P_LT_ (W/kg)	1.77 ± 0.29	2.82 ± 0.44 *	<0.001	2.88	-
SmO_2 2MMOL_ (%)	52.2 ± 13.1	48.8 ± 11.2	0.49	0.27	-
SmO_2AT4_ (%)	45.2 ± 11.1	32.7 ± 11.5 *	<0.001	-	0.68
SmO_2DMAX_ (%)	44.3 ± 10.3	31.0 ± 9.2 *	<0.001	-	0.82
SmO_2LT_ (%)	45.6 ± 10.5	33.9 ± 9.6 *	0.007	-	0.66
**Males**					
**Variable/Group**	**Students** **(n = 16)**	**Speed Skaters (n = 14)**	***p*** **-Value**	**Effect Size (Females)**	
				**Cohen’s d**	**Rank Biserial** **Correlation (r)**
P_2 MMOL_ (W)	103 ± 28	201 ± 35 *	<0.001	2.64	-
P_2 MMOL_ (W/kg)	1.25 ± 0.43	2.72 ± 0.57 *	<0.001	2.95	-
P_AT4_ (W)	173 ± 27	267 ± 41 *	<0.001	2.78	-
P_AT4_ (W/kg)	2.08 ± 0.40	3.62 ± 0.46 *	<0.001	3.58	-
P_DMAX_ (W)	173 ± 22	263 ± 43 *	<0.001	2.67	-
P_DMAX_ (W/kg)	2.08 ± 0.33	3.56 ± 0.44 *	<0.001	-	0.98
P_LT_ (W)	177 ± 22	253 ± 48 *	<0.001	-	0.96
P_LT_ (W/kg)	2.13 ± 0.29	3.41 ± 0.48 *	<0.001	-	1.0
SmO_2 2MMOL_ (%)	45.0 ± 13.2	42.5 ± 7.7	0.54	0.22	-
SmO_2AT4_ (%)	30.5 ± 15.5	28.3 ± 8.6	0.64	0.17	-
SmO_2DMAX_ (%)	32.5 ± 13.3	29.4 ± 6.5	0.44	0.29	-
SmO_2LT_ (%)	31.8 ± 13.1	30.4 ± 7.1	0.71	0.14	-

P_2 MMOL_—power at aerobic threshold (lactate = 2 mmol/L); P_AT4_—power at anaerobic threshold (lactate = 4 mmol/L); P_DMAX_—power at modified Dmax threshold; P_LT_—power at lactate threshold; SmO_2 MMOL_—muscle oxygenation threshold (lactate = 2 mmol/L); SmO_2 AT4—_muscle oxygenation threshold (lactate = 4 mmol/L); SmO_2DMAX_—muscle oxygenation Dmax threshold; SmO_2LT_—muscle oxygenation at lactate threshold; *—difference (*p* < 0.05) between female students and female speed skaters, or male students and male speed skaters.

## Data Availability

The authors confirm that the data supporting the findings of this study are available within the article and will be made available from the corresponding author on reasonable request. The data (Table 1 and Table 2) of the presented study of highly trained female and male speed skaters were partially used in the following publication: Rębiś K., D. Sadowska, M. Starczewski, A. Klusiewicz. Usefulness of portable device to establish differences in muscle oxygenation between the Wingate test and graded exercise test: effect of gender on anaerobic and aerobic capacity in speed skaters. Front. Physiol. 2022, 13, 809864. doi: 10.3389/fphys.2022.809864 [13].
